# Allostatic Load and Metabolic Syndrome in Depressed Patients: A Cross-Sectional Analysis

**DOI:** 10.1155/2024/1355340

**Published:** 2024-06-06

**Authors:** Francis Osei, Pia-Maria Wippert, Andrea Block

**Affiliations:** ^1^Professorship for Medical Sociology and Psychobiology, Department of Health and Physical Activity, University of Potsdam, Potsdam 14469, Germany; ^2^Faculty of Health Sciences, joint Faculty of the University of Potsdam, The Brandenburg Medical School Theodor Fontane, and the Brandenburg University of Technology Cottbus – Senftenberg, Am Mühlenberg 9 14476, Potsdam, Germany

## Abstract

Allostatic load (AL) is the cumulative wear and tear on the body due to the chronic adverse physical or psychosocial situations. The acute stress response activates the primary mediators of AL, which include cortisol, epinephrine (EPI), norepinephrine (NE), and dehydroepiandrosterone sulfate (DHEA-S). Secondary outcomes, such as metabolic syndrome (MetS), cardiovascular, and immune system changes, can result from long-term stress responses. Given these complex reactions to an acute stressor, a multidimensional stress assessment is required when investigating individual stress reactivity in an experimental setting. This study is aimed at examining the association between the primary mediators of AL and MetS in major depressive disorder (MDD) patients. MDD patients (*n* = 164, age = 18–65 years old) with MetS+ (*n* = 46, weight = 93.10 ± 16.43 kg) and without MetS- (*n* = 118, weight = 73.08 ± 15.22 kg) were analyzed cross-sectionally. Stepwise binary regression and Welch's *t*-test were used to find the associations and differences between the two groups. The regression analysis was fully adjusted for age, sex, and the Beck Depression Inventory-II score. In unadjusted model, cortisol (*b* = −0.003, *p* = 0.034) was inversely associated with MetS. In fully adjusted model, EPI (*b* = −0.006, *p* = 0.007) was inversely associated with MetS. However, significant differences (*p* = 0.005) were observed for cortisol between MDD patients without MetS- (410.13 ± 144.63 nmol/l) and MDD patients with MetS+ (340.90 ± 132.98 nmol/l) with a small effect size (Cohen's *d* of 0.489). Significant differences (*p* = 0.001) were observed for EPI between MDD patients without MetS- (185.67 ± 124.44 pg/ml) and MDD patients with MetS+ (124.95 ± 84.38 pg/ml) with a moderate effect size (Cohen's *d* of 0.530). These observations are of clinical importance for the management of MDD patients.

## 1. Introduction

Allostasis is defined as a state of adaptive responsiveness to adversity, whereby the autonomic nervous system (ANS), the hypothalamic-pituitary-adrenal (HPA) axis, the hypothalamic-pituitary-thyroid (HPT) axis, the hypothalamic-pituitary-gonadal (HPG) axis, the hypothalamic-pituitary-somatotropic (HPS) axis, and the hypothalamic-pituitary-prolactin (HPP) axis as well as the immune system are involved in promoting physiological stability [1–3]. However, the cumulative wear and tear on the body due to adapting to chronic adverse physical or psychosocial situations is known as allostatic load (AL) [4]. Immediately after stress onset, activation of the sympathetic nervous system (SNS) triggers the production of catecholamines (i.e., epinephrine (EPI), norepinephrine (NE), and dopamine) in the adrenal medulla [5]. In parallel, activity of the slower-acting HPA axis increases. As a consequence of a multistage cascade, a rise in corticosteroids, i.e., cortisol, can be observed by analyzing saliva samples with a peak at about 20−30 minutes after the onset of a typical laboratory stress paradigm (e.g., Trier Social Stress Test (TSST)) [6]. On a neural level, catecholamines and corticosteroids interact with cortical and subcortical structures, contributing to a strategic resource reallocation in the face of acute stress. Regions associated with the salience network, e.g., the anterior insula, the dorsoanterior cingulate cortex (dACC), and the amygdala [7], show increased activity following stressful events, whereas the prefrontal cortex shows decreased activity in stressful conditions [8]. These dynamic neuroendocrine interactions orchestrate a complex psychophysiological response to stress to enable rapid and adequate reactions to a changing environment [9]. In addition to these rapid endocrine stress effects, cortisol exerts inner-cellular genomic effects due to its lipophilic character, lasting from hours to days or even months after acute stress exposure [10, 11]. Given these complex reactions to an acute stressor, a multidimensional stress assessment is required when investigating individual stress reactivity in an experimental setting.

In the second stage, the stress response causes anti-inflammation due to cortisol release, leading to the inhibition of nuclear factor kappa B (NF*κ*B) and decreasing cytokine levels [12, 13]. Additionally, a more long-term stress response results in secondary outcomes, which can be transferred by an altered HPA axis. Altered HPA axis can lead to changes in metabolic (e.g., metabolic syndrome (MetS)), cardiovascular (e.g., systolic and diastolic blood pressure and heart rate) [14], and immune systems (e.g., C-reactive protein (CRP) and fibrinogen). Tertiary outcomes are related to alterations on all hypothalamic axes and include poor health, mental/cognitive decline (e.g., loss of brain function and mental disorders), or degenerative diseases up to the exhaustion of organ systems [15–17].

In stress neurobiology, stress mediators work in a feedback loop to accurately regulate the HPA axis and sympathetic adrenal medullary (SAM) system to regain homeostasis after disappearing of the stressor [5]. The stress responsive systems are interrelated, and the activation of the HPA axis is aided by stress-induced NE production in certain brain areas such as the locus coeruleus, hippocampus, and amygdala [18, 19]. Acute and chronic stress alters the regulation of the HPA axis and autonomic responses via various routes [20, 21]. In the presence of chronic stress, neurochemical data shows that the HPA axis and SAM system excitability remain elevated, as does the locus coeruleus sensitivity to corticotrophin-releasing hormone (CRH) and the enzyme tyrosine hydroxylase, which increases NE production (i.e., CRH–NE loop) [22]. Some of these alterations may be related to the loss of pituitary glucocorticoid self-regulation and the paraventricular nucleus of the hypothalamus. When the glucocorticoid negative feedback on the HPA axis is disrupted, it remains activated, resulting in the persistence of increased systemic glucocorticoid levels [23]. This can cause metabolic illnesses and immunosuppression and contribute to the development of autoimmune diseases and mental disorders [24].

Major depressive disorder (MDD) is a global health issue [25]. A recent systematic review and meta-analysis on MDD, involving twenty studies and *n* = 18,953 adult participants, found a global prevalence rate of 13.3% (CI: 8.4-20.3%) [26]. Interestingly, there have been several reports showing the linkage between the primary mediators of AL (i.e., cortisol, NE, EPI, and DHEA-S) and MDD [27–29]. MDD is associated with HPA axis dysfunction, partly resulting from insufficient glucocorticoid negative feedback loops due to chronic stress [30–32]. Precisely, chronic activation of the primary mediators of AL impairs both the HPA axis and SAM function, which leads to MDD [33]. Furthermore, chronic high glucocorticoid levels enhance gluconeogenesis, leading to insulin resistance, obesity, and MetS development [27, 34]. Additionally, the anhedonia feature of MDD may foster unhealthy eating, weight gain, and visceral fat accumulation [35]. Weight gain, obesity, and visceral fat accumulation are precursors for MetS development in MDD patients [36]. This is partly due to the triggering of low-grade inflammation resulting from monoaminergic and neurotrophic system changes in metabolic and redox function from HPA axis dysfunction [37]. Thus, there is a cascade of physiological changes affecting the HPA axis and SAM function in MDD and MetS pathology.

MetS is defined as the cluster of coexistence of high blood pressure, abdominal obesity, low high-density lipoprotein (HDL) cholesterol, elevated triglycerides, and hyperglycemia [38]. MetS and metabolic abnormalities have been reported in individuals affected by MDD [29, 39, 40]. Vancampfort et al. [41] reported that patients with MDD had a higher prevalence of MetS (29.7%), hyperglycemia (18.8%), and hypertriglyceridemia (30.1%) when compared to age- and gender-matched controls. Moreover, Almadi et al. [42] reported an association between cortisol and MetS in workers who reported being stressed. Interestingly, patients with an increasing number of components of MetS show higher 24-hour urinary NE excretion [43]. Moreover, associations between higher sympathetic neural drive, hypertension, and MetS have been reported in individuals with psychosocial stress [44]. Additionally, DHEA-S has been reported to play a role in the prevention and treatment of MetS [45], as it exerts beneficial effects by stimulating lipolysis [46], reducing overeating via blockage of neuropeptide Y (NPY) [47], and reducing human visceral fat accumulation via upregulation of adiponectin gene expression [48]. Nevertheless, low levels of DHEA-S have been found in individuals affected by depression and MetS [28, 49].

Osei et al. [28] reported an association between the primary mediators of AL and MetS in a systematic review. However, this systematic review focuses on primary mediators of AL and MetS and did not involve clinical studies with patients affected by MDD. In vulnerable populations like MDD patients, understanding how the primary mediators of AL may affect the neuroendocrine system could provide insights that are crucial for clinical management. Also, limited studies exist on the primary mediators of AL and MetS in MDD patients [50, 51]. Hence, the aim of this study is to examine if there is an association between the primary mediators of AL and MetS in depressed patients to foster preventive strategies for this frequent comorbidity.

## 2. Materials and Methods

### 2.1. Study Population

For this study, data from the prospective, controlled observational study DEPREHA were used for a secondary analysis. The DEPREHA study was conducted between January 2015 and December 2017. The DEPREHA study was an 18-month multicentre study with *n* = 240 depressive patients (age = 18–65 years, females = 79.9%) treated in the settings of a psychosomatic rehabilitation clinic, an outpatient psychiatric practice, and a psychotherapeutic outpatient clinic [52]. The patients were diagnosed by the physicians and therapists at the clinic (see inclusion criteria). The Beck Depression Inventory-II (BDI-II) was additionally measured [53]. Inclusion criteria were adults 18–65 years of age, >21 days of absenteeism within the last 12 months, depressive episode (ICD-10, F32.x or F33.x), dysthymia (F34.1), or adjustment disorder with prolonged depressive reaction (F43.21). Exclusion criteria included pregnancy, hormone therapy (excluding oral contraceptives or thyroid medication), inability to fill out a questionnaire, intellectual disability, or severe diseases like neurological, psychotic, personality disorders, or substance disorders (for details, see [52]). For the presented study, *n* = 164 complete datasets (questionnaires and biomarkers from baseline measurement (t0) at entrance to treatment settings) out of *n* = 240 (total sample) were used. Besides standardized questionnaires, demographic characteristics as well as medication intake were assessed.

### 2.2. Ethical Approval

All clinical investigations were conducted according to the principles expressed in the Declaration of Helsinki of 1975, as revised in 2013. All participants were fully informed, in written and verbal form, about the intent and content of the study. All participants gave written informed consent. The final ethical approval was provided by the major institutional ethics review board of the University of Potsdam, Germany (number 13/15).

### 2.3. Measurement of Depression

The Beck Depression Inventory-II (BDI-II) was used to assess depressive symptoms and severity [53]. The BDI-II is a 21-item self-report questionnaire that addresses current affective, cognitive, motivational, and physiological symptoms of depression. The BDI-II has been shown to measure non-cognitive factors (i.e., somatic affective symptoms), usually represented by loss of energy and irritability, and cognitive factors (i.e., psychological symptoms), such as self-dislike and worthlessness [54]. In Germany, the BDI-II has been validated in both clinical and non-clinical samples with an internal consistency of Cronbach's alpha of 0.84–0.93 and retest reliability of *r* = 0.75–0.92 [55, 56].

### 2.4. MetS Diagnosis

MetS was diagnosed based on the definition by Alberti et al. [38]. Instead of waist circumference, body mass index (BMI ≥ 25 kg/m^2^) was used as a criterion for MetS in accordance with the guidelines of the American Association of Clinical Endocrinologists [57] (see Table 1 for details). Information concerning medication taken by participants was available from medical records. The presence of any three of the five defined risk factors in Table 1 constitutes a diagnosis of MetS.

### 2.5. Anthropometric Measurements

Weight and height were measured by health care professionals upon admission to the primary care setting, and data were available from medical records. The ratio of weight (kg) to height (m^2^) was calculated for BMI.

### 2.6. Primary Mediators of Allostatic Load

In the DEPREHA project, twenty-one allostatic load mediators were assessed based on the concept of AL and its influence on psychosomatic disorders [2, 58]. The twenty-one mediators of AL consisted of glucose metabolic biomarkers (i.e., glycosylated haemoglobin (HbA1c) and plasma glucose, fasting insulin, and BMI), lipid metabolic biomarkers (i.e., triglycerides, HDL-C, low-density lipoprotein density cholesterol (LDL-C), and total cholesterol), sympathetic nervous system biomarkers (i.e., plasma EPI, NE, and dopamine), hypothalamus-pituitary-adrenal (HPA) axis (i.e., serum-cortisol and serum-DHEA-S, serum cortisol, and serum-aldosterone), immune system (i.e., soluble E-selectin, soluble intercellular adhesion molecule-1 and TNF-*α*, interleukin-6, and fibrinogen), and cardiovascular markers (i.e., systolic and diastolic blood pressure and heart rate). In the current study, the primary mediators of AL consisting of cortisol, DHEA-S, EPI, and NE were utilized. Primary mediators of ALI scores were calculated as the sum of single biomarkers falling within the high-risk quartile of the sample.

### 2.7. Laboratory Diagnostic Criterion Measurements

Blood samples were drawn (morning hours, 7–9 am) from the arm by trained health professionals and collected in plain blood collection tubes or tubes containing EDTA, citrate, or sodium fluoride for subsequent analysis at the diagnostic lab at the Ernst von Bergmann Clinic, Potsdam, Germany. HDL-C, LDL-C, cholesterol, and triglycerides were assessed using Roche/Hitachi Cobas 701/702 (F. Hoffmann-La Roche, Ltd., Basel, Switzerland) via enzymatic colorimetric assays. Fasting plasma glucose was measured via a hexokinase enzyme reaction using the Roche Cobas 8000 (Roche Diagnostics Ltd., Basel, Switzerland).

HPA axis biomarkers such as serum cortisol and serum DHEA-S were measured and analyzed by immunological in vitro test electrochemiluminescence immune assay (REF 06687733 190 for serum cortisol, REF 03000087 for serum DHEA-S; both Roche Cobas, Modular Analytics E17, F. Hoffmann-La Roche, Ltd., Investor Relations Basel, Switzerland). Sympathetic nervous system (SNS) biomarkers such as EPI and NE were measured in plasma, and analysis was performed with enzyme-linked immunosorbent assays (ELISA) (RE59251 for EPI and RE59261 for NE; both from IBL International GmbH, Hamburg, Germany).

### 2.8. Statistical Analysis

All data were entered into the Statistical Package for Social Science (SPSS 27.0, Chicago, IL, USA) computer software for analysis. The Kolmogorov-Smirnov test for normality was performed. The data was evenly distributed. Descriptive statistics are presented as mean and standard deviation for metric variables and frequency (*n*) and percentage (%) for ordinal or nominal variables. Due to the unequal sample sizes between MDD patients with MetS+ and MDD patients without MetS-, Welch's *t*-test was performed to find the differences between the two groups on continuous variables, and Cohen's *d* was used as an effect size estimate. Chi-square tests were performed on categorical variables and Fisher's exact test to analyze categorical variables between MDD patients with MetS+ and without MetS-. The level of significance was set at *p* < 0.05. A stepwise binary regression was performed to analyze the association between the primary mediators of AL and MetS. Further adjustments were made for age, sex, and BDI-II score because evidence shows that these confounders influence depression diagnosis and MetS [39, 59, 60] and may have an impact on the association between the primary mediators of AL and MetS. To adjust for multiple comparisons, a Bonferroni correction was applied which decreased the significance level to aBonf = alpha/5 = 0.01.

## 3. Results

### 3.1. Study Characteristics

There was *n* = 131 (79.9%) women and *n* = 33 (20.1%) men with clinically diagnosed depression available from the DEPREHA sample (*n* = 164, 100.0%). Patients with MetS+ (*n* = 46, 28%) were slightly older and heavier than patients without MetS- (*n* = 118, 72.0%) (see Tables 2 and 3). In accordance with the diagnosis of MetS, metabolic biomarkers were higher in MDD patients with MetS+ than MDD patients without MetS-, except for HDL-C. BDI-II scores for MDD patients without MetS- were slightly higher (23.86 ± 10.49) than MDD patients with MetS+ (22.42 ± 8.93).

MDD patients with MetS+ and without MetS- were both taking antidepressant medications. Lower levels of cortisol and DHEA-S were observed in MDD patients with MetS+ as compared to MDD patients without MetS-. Higher levels of EPI were observed in MDD patients without MetS- as compared to MDD patients with MetS+. Levels of NE in MDD patients with MetS+ were similar to those in MDD patients without MetS-. See Tables 2 and 3 for all study variables.

### 3.2. Comparison between Depressive Patients with Metabolic Syndrome and without Metabolic Syndrome

Results from Welch's *t*-test indicated significant difference (*p* = 0.005) in the mean test scores on cortisol between MDD patients with MetS+ (340.90 ± 132.98 nmol/l) and MDD patients without MetS- (410.13 ± 144.63 nmol/l), with a small effect size (Cohen's *d* of 0.489). The mean test scores for EPI were higher in the MDD patients without MetS- (185.67 ± 124.44 pg/ml) than the MDD patients with MetS+ (EPI = 124.95 ± 84.38 pg/ml). This was a significant difference (*p* = 0.001) with a moderate effect size (Cohen's *d* of 0.530).

However, no significant differences were found on DHEA-S (Cohen's *d* = 0.213, *p* = 0.23), NE (Cohen's *d* = 0.002, *p* = 0.993), and primary ALI (Cohen's *d* = 0.233, *p* = 0.210) between MDD patients with MetS+ and MDD patients without MetS- (see Table 3).

### 3.3. Association between Depressive Patients with Metabolic Syndrome and without Metabolic Syndrome

In the stepwise binary regression analysis, cortisol showed a significant inverse association with MetS (*b* = −0.004, OR = 0.996, [95% CI =0.994–0.996], *p* = 0.008) in the unadjusted model. This shows that for each 10 nmol/l serum cortisol increase, the probability of having MetS is reduced by 4%. The results were similar after adjusting for age (*b* = −0.003, OR = 0.997, [95% CI =0.994–0.999], *p* = 0.018). No significant associations (*p* > 0.01) between cortisol and MetS were observed after adjusting for age and sex and as well as in the fully adjusted model (i.e., age, sex, and BDI-II score).

Moreover, the binary regression analysis revealed that EPI has a significant inverse association with MetS (*b* = −0.005, OR = 0.995, [95% CI = 0.991–0.999], *p* = 0.007) in the unadjusted model. This suggests that the probability of not having a MetS is reduced for each 10 pg/ml plasma EPI increase measured with ELISA by about 5%. Similar results were seen after adjusting for age (*b* = −0.005, *p* = 0.006) and likewise age and sex (*b* = −0.005, *p* = 0.008). After fully adjusting for age, sex, and BDI-II score, EPI was still inversely associated with MetS (*b* = −0.006, OR = 0.994, [95% CI = 0.990–0.998], *p* = 0.007). This suggests that the probability of not having a MetS is reduced for each 10 pg/ml plasma EPI increase measured with ELISA by about 6%. In contrast, no significant associations (*p* > 0.01) were observed for DHEA-S, NE, and primary ALI with MetS in both unadjusted and adjusted models. Details are outlined in Table 4.

## 4. Discussion

One major finding from the study was that cortisol was inversely associated with MetS, even after adjusting for age. But this association was not evident after adjusting for age and sex and in the fully adjusted model (i.e., age, sex, and BDI-II score). This result is in contrast with previous studies that reported a positive association between cortisol and MetS [61, 62]. Interestingly, a meta-analysis on MDD patients (*n* = 5,531) showed greater rates of MetS (29.7%), hyperglycemia (18.8%), and hypertriglyceridemia (30.1%) than age- and gender-matched controls [41]. However, another meta-analysis of a population of *n* = 11, 808 subjects found no significant association between cortisol and MetS [63]. It should be noted that factors such as population base, age, gender, assessment method, and study design play a significant role in cortisol levels and its association to MetS [63].

Despite this, cumulative stress leading to chronic stress may alter HPA axis secretion and function and thus increase glucocorticoids [1, 4, 64]. During acute stress, HPA axis activity is stimulated by CRH neurons located in the paraventricular nucleus (PVN) of the hypothalamus [33]. The stressors then stimulate the release of CRH into the hypophysial portal vessels to cause the transportation of peptides to the anterior pituitary, which permits corticotrophs entry. This intends to stimulate corticotrophs to enhance the release of adrenocorticotropic hormone (ACTH) into the systemic circulation. ACTH then fosters the secretion of glucocorticoids in the adrenal cortex [33, 65]. In addition, the *α*1 and *α*2 adrenoreceptors of EPI and NE enhance CRH and ACTH during both acute and chronic stress challenges [66]. Besides, in chronic stress, both glucocorticoid negative feedback and CRH-NE loop activation led to HPA axis disruption. HPA axis disruption can trigger metabolic diseases, immunosuppression, and mental problems [22–24].

Also, MDD patients without MetS- had higher levels of serum cortisol compared to MDD patients with MetS+ in this current study. This can be explained from two perspectives. On one hand, hypercortisolaemia is associated with MDD, and perhaps not all MDD patients with hypercortisolaemia may have MetS or metabolic abnormalities. Cortisol's lipophilic nature causes inner-cellular genomic effects that can persist for hours, days, or even months after acute stress exposure [10, 11]. Also, chronic stress induces hypercortisolaemia, which then leads to low-grade inflammation [67]. Moreover, hypercortisolaemia may disrupt HPA axis function and, together with alterations in both monoaminergic and neurotrophic systems, impair metabolic and redox status and enhance low-grade inflammation in MDD [37]. Low-grade inflammation could take several months to years before the development of MetS [68, 69]. Also, chronic stress could lead to MDD due to desensibilization of the glucocorticoid receptor resulting from high levels of cortisol [23, 30–32]. Higher levels of cortisol may promote gluconeogenesis and liberate free fatty acid (FFA) efflux from adipocytes into the bloodstream, leading to central fat accumulation and MetS development [27, 34, 70, 71]. On the other hand, the extended period of cortisol release resulting from chronic stress can lead to HPA axis overactivity [72, 73]. HPA axis overactivity can lead to low HPA axis activity which may be due to adrenal dysfunction or adrenal fatigue. This state of low HPA axis activity is often influenced by increased negative feedback sensitivity of the HPA axis leading to hypocortisolism [72]. Maripuu et al. [73] found an association between hypocortisolism and MetS in 245 patients with recurrent or bipolar depression.

Moreover, the results suggest an inverse association between EPI and MetS; more specifically, MDD patients without MetS- had higher levels of EPI than MDD patients with MetS+ in this current study. Both EPI and NE are stress hormones activated in both acute stress and chronic stress [74, 75]. High levels of EPI are a sign of a higher sympathetic neural drive and are evident in MetS and individuals with psychosocial stress [44, 76]. The results further revealed no association between NE and MetS. However, psychological stress has been reported to enhance the development of MetS via the release of EPI and NE [74]. In addition, increasing numbers of components of MetS in patients have been shown to excrete higher 24-hour urinary NE [43]. Interestingly, the mean NE values between MDD patients with MetS+ and MDD patients without MetS- patients were comparable. From the demographics of the study participants, it should be noted that the participants were on different medications. Drugs such as beta-blockers bind to beta-adrenoceptors and prevent EPI and NE from binding to these receptors. This may alter the function of SNS [77].

Furthermore, no significant association was observed for DHEA-S and MetS. Also, the present study showed that MDD patients with MetS+ do not differ in DHEA-S levels compared to MDD patients without MetS-. However, previous studies have confirmed that lower levels of DHEA-S are associated with MetS and depression [28, 49]. DHEA-S levels decline with age and are a risk factor for developing MetS [78].

The study revealed no significant association between primary ALI and MetS. In this study, the primary mediators (i.e., cortisol, DHEA-S, EPI, and NE) for ALI calculation were derived from Seeman et al. [17]. On the contrary, there are other combinations of mediators (i.e., aldosterone, dopamine, fibrinogen, interleukin-6, IGF-1, CRP, TNF-*α*, and DHEA) that are used in calculating ALI for the primary mediators of AL by other authors [14, 79, 80]. These different combinations of mediators that are used in calculating ALI for the primary mediators of AL may yield different associations with MetS. Precisely, acute stress affects the ANS and SAM systems due to the release of cytokines (CRP, interleukin-6, interleukin-1*β*, and tumor necrosis factor alpha (TNF-*α*)) [75, 81]. Also, interleukin-6, TNF-*α*, and CRP have been reported to be associated with MDD and MetS [82, 83]. The physiological mediators are reciprocal, linked, and exhibit non-linear effects during the allostasis-adaptation process [64, 84]. Hence, future research is recommended to understand the set of primary mediators of AL in calculating ALI that yield the best association with MetS.

The results additionally revealed that both MDD patients with MetS+ and without MetS- do not differ in the prevalence of antidepressant medication and subtypes of depression (i.e., single and recurrent episodes). Notwithstanding, antidepressant usage has been reported to alter HDL-C in the short term [85]. Tricyclic drugs lead to higher metabolic and inflammatory alterations than their selective serotonin reuptake inhibitor (SSRI) counterparts [86]. Additionally, higher levels of plasma EPI and increased heart rate have been reported in MDD patients taking SSRI antidepressant drugs [87, 88]. On the contrary, the long-term effects of antidepressant usage on both lipid and metabolic biomarkers in MDD patients are limited and with inconsistent results [89–91]. Thus, more research is needed to understand how antidepressant usage affects MetS status in MDD patients.

In this study, MDD patients with MetS+ had a higher mean age than MDD patients without MetS-. It should be noted that age-related changes may lead to neuroendocrine alterations causing MetS and MDD [29, 39, 40]. Another major finding was that MDD patients with MetS+ had a high body mass index (BMI = 31.9 ± 5.71 kg/m^2^), which corresponds to obesity. This is in line with research that found depression is associated with both obesity and MetS, independently of one another [92]. In contrast, the results revealed that MDD patients without MetS- also had a high body mass index (BMI = 25.62 ± 4.79 kg/m^2^), which corresponds to being overweight. Interestingly, according to the research by Tomiyama et al. [93] with *n* = 40,420 participants, 29% of obese people were metabolically healthy, whereas 30% of those with normal body weight were classified as cardiometabolically unhealthy. However, Bremner et al. [36] reported that stress can operate on the brain and cause an increase in overeating and overweight, which can lead to MetS and MDD. This is because the anhedonia feature of MDD leads to an unhealthy lifestyle, resulting in visceral fat accumulation [35]. Nevertheless, the pathophysiology of MDD and MetS is complex. Notably, chronic stress has complicated and long-term effects on the neuroendocrine system. Thus, future studies focusing on longitudinal data are warranted to understand the association of the primary mediators of AL and MetS in MDD patients.

### 4.1. Strengths and Limitations

This study is one of the few studies [50, 51] reporting on the association between the primary mediators of AL and MetS in MDD patients. The study has added novel contributions from the neuroendocrine perspective of MetS in MDD patients. The study shows that cortisol and EPI have an inverse association with MetS. The study also shows that both cortisol and EPI are elevated in MDD patients without MetS-. Hence, this study is vital for the clinical management of MDD patients. Despite these strengths, there are limitations to be considered. Firstly, the cross-sectional nature of the data analysis prohibits any causal interpretations of the revealed correlation between the primary mediators of AL and MetS in MDD patients. Future studies employing a longitudinal follow-up research design are recommended to overcome this shortcoming of the current analysis. Secondly, the study lacked information on the type of antidepressant medication, the duration of antidepressant medications, or whether there was a switch in antidepressant medication. A switch in antidepressant medications alters EPI, NE, and lipid markers [77, 85]. Thirdly, nutritional intake and physical activity, which influence the primary mediators of AL and the biomarkers used in the diagnosis of MetS in MDD patients, were not accounted for in the current study.

Also, the baseline sample size was sufficient, but missing data may have impacted the results, limiting generalization. Cortisol production follows a diurnal rhythm, with a significant rise in the first hour after awakening known as the cortisol awakening response (CAR). During the CAR period, cortisol levels can rise between 38 and 75%, peaking around 30 and 45 minutes after waking [94]. This shows that CAR levels can be altered based on the phase of the endogenous circadian cycle at which samples are assessed [95]. Although MDD patients without MetS had higher cortisol levels than MDD patients with MetS+, the cortisol levels were all in the normal ranges (morning reference range (6 : 00–10 : 30 h) = 127–497 nmol/l) [96]. Interestingly, no significant differences were seen between time of blood assessment between MDD patients with MetS+ and MDD patients without MetS- in the current study. Notably, CAR has been shown to differ between MDD patients [97–99]. However, time of sleep, time of wake, and sleep/wake ratio, which can affect CAR, were not accounted for in this study. Foremost, using liquid chromatography with tandem mass spectrometry to measure 24 h urinary cortisol, EPI, and NE is the gold standard [100, 101]. Changes in albumin or cortisol-binding globulin levels in serum cortisol may not reflect unbound (free) cortisol levels [100]. Further research considering the factors mentioned above could unravel approaches for understanding the association between the primary mediators of AL and MetS in MDD patients.

## 5. Conclusions

In summary, this study indicates that cortisol and EPI may have an inverse association with MetS in MDD patients. MDD patients without MetS- showed increased cortisol and EPI levels. However, MDD patients with MetS+ showed reduced cortisol levels which may be due to increased negative feedback sensitivity of the HPA axis and adrenal dysfunction or adrenal fatigue. These observations are of clinical importance for the management of MDD patients. Future studies focusing on longitudinal data are warranted for clarification of these results and to understand the association between the primary mediators of AL and MetS in MDD patients.

## Figures and Tables

**Table 1:** Diagnostic criteria for MetS. The presence of 3 any of the following 5 risk factors constitute a diagnosis of MetS.

Measure	Cut points/criteria
Elevated blood pressure	Systolic ≥ 130 mmHg and/or diastolic ≥ 85 mmHg (or antihypertensive drug treatment)
Elevated triglycerides	≥ 150 mg/dl (or drug treatment for elevated triglycerides)
Reduced high density lipoprotein cholesterol (HDL-C)	Males: < 40 mg/dl; females: < 50 mg/dl (or drug treatment for reduced HDL-cholesterol)
Elevated blood glucose	≥ 100 mg/dl (fasting) (or drug treatment for elevated glucose/type 2 diabetes mellitus)
Elevated body mass index (BMI)	BMI ≥ 25 kg/m^2^⁣^∗^

⁣^∗^Strazzullo et al. [57].

**Table 2:** Study characteristics of MDD patients with metabolic syndrome and without metabolic syndrome.

Variables	Sample		Missing data		MetS+				MetS-				Test (df)	Cohen's *d*	*p*
	*N*	%	*N*	%	*N*	%	*M*	±SD	*N*	%	*M*	±SD			
Age	164	100	0	0.0	46	28.0	51.17	9.28	118	72.0	47.07	9.97	*t* = −2.492 (87.714)	-0.420	0.015
Sex													*χ* ^2^ = 6.021 (1)	-	0.013
Women	131	79.9	-	-	31	18.9	-	-	100	61.0	-	-	-	-	
Men	33	20.1	-	-	15	9.1	-	-	18	11.0	-	-	-	-	
Academic traning	162	98.7	-	-	2	1.3	-	-	116	70.7	-	-	*χ* ^2^ = 6.072 (7)	-	0.531
No training	9	5.4	-	-	4	2.4	-	-	5	3.0	-	-	-	-	
Student	3	1.8	-	-	1	0.6	-	-	2	1.2	-	-	-	-	
Apprenticeship	57	35.0	-	-	15	9.2	-	-	42	25.5	-	-	-	-	
Vocational school	15	9.0	-	-	7	4.3	-	-	8	4.9	-	-	-	-	
Other vocational school	5	3.1	-	-	2	1.2	-	-	3	1.8	-	-	-	-	
Technical school	32	19.5	-	-	9	5.5	-	-	23	14.0	-	-	-	-	
University of applied sciences	16	9.7	-	-	4	2.4	-	-	12	7.3	-	-	-	-	
University	25	15.2	-	-	4	2.4	-	-	21	13.0	-	-	-	-	
Family status	164	100	0	0.0	46	28.0	-	-	119	72.0	-	-	*χ* ^2^ = 3.218 (5)	-	0.666
Single	34	20.7	-	-	7	4.3	-	-	27	16.4	-	-	-	-	
Married	78	47.6	-	-	26	15.8	-	-	52	32.0	-	-	-	-	
Partnership	20	12.2	-	-	6	3.7	-	-	14	8.5	-	-	-	-	
Widowed	6	3.7	-	-	2	1.2	-	-	4	2.4	-	-	-	-	
Divorced	22	13.4	-	-	4	2.4	-	-	18	10.9	-	-	-	-	
Living separately	4	2.4	-	-	1	0.6	-	-	3	1.8	-	-	-	-	
Net income (€)	156	95.1	8	4.9	45	27.4	-	-	111	67.7	-	-	*χ* ^2^ = 3.858 (7)	-	0.796
Under 1250	26	15.9	-	-	6	3.7	-	-	20	12.2	-	-	-	-	
1250–1749	39	23.7	-	-	13	7.9	-	-	26	15.9	-	-	-	-	
1750–2249	23	14.0	-	-	7	4.3	-	-	16	9.8	-	-	-	-	
2250–2999	27	16.5	-	-	10	6.0	-	-	17	10.4	-	-	-	-	
3000−3900	18	11.0	-	-	3	1.8	-	-	15	9.1	-	-	-	-	
4000–4900	17	10.4	-	-	5	3.1	-	-	12	7.3	-	-	-	-	
Over 5000	2	1.2	-	-	0	0	-	-	2	1.2	-	-	-	-	
Undisclosed	4	2.4	-	-	1	0.6	-	-	3	1.8	-	-	-	-	
Depression (BDI-II score)	155	94.5	9	5.5	43	26.2	22.42	8.93	112	68.3	23.86	10.49	*t* = 0.856 (88.926)	0.394	0.143
Depression type	120	73.2	44	26.8	41	25.0	-	-	79	48.2	-	-	*χ* ^2^ = 2.999 (2)	-	0.233
Single episode	65	39.6	-	-	25	15.2	-	-	40	24.4	-	-	-	-	
Recurrent	37	22.6	-	-	13	8.0	-	-	24	14.6	-	-	-	-	
Not applicable	18	11.0	-	-	3	1.8	-	-	15	9.2	-	-	-	-	
Depression severity	155	94.5	9	5.5	43	26.2	-	-	112	68.3	-	-	*χ* ^2^ = 4.413 (4)	-	0.353
None	9	5.5	-	-	2	1.2	-	-	7	4.3	-	-	-	-	
Minimal	19	11.6	-	-	4	2.4	-	-	15	9.1	-	-	-	-	
Mild	25	15.2	-	-	9	5.5	-	-	16	9.8	-	-	-	-	
Moderate	55	33.5	-	-	19	11.6	-	-	36	22.0	-	-	-	-	
Severe	47	28.7	-	-	9	5.5	-	-	38	23.1	-	-	-	-	
Antidepressant medication	147	89.6	17	10.4	41	24.9	-	-	106	64.7	-	-	*χ* ^2^ = 551 (1)	-	0.458
Yes	116	70.7	-	-	34	20.7	-	-	82	50.0	-	-	-	-	
No	31	18.9	-	-	7	4.2	-	-	24	14.7	-	-	-	-	

Key: BDI-II score: Beck Depression Inventory-II score. Significant at *p* < 0.05.

**Table 3:** Anthropometric data and biomarkers in depressed patients with metabolic syndrome and without metabolic syndrome.

Variables	Sample		Missing Data		MetS+				MetS-				Test (df)	Cohen's (*d*)	*p*
	*N*	%	*N*	%	*N*	%	*M*	±SD	*N*	%	*M*	±SD			
Anthropometry															
Weight (kg)	160	97.5	4	2.6	46	28.0	93.10	16.43	114	69.5	73.08	15.22	*t* = −7.128 (77.837)	-1.286	**0.001**
Height (cm)	159	96.9	5	3.1	46	28.0	171.02	8.22	113	68.9	168.59	8.31	*t* = −1.685 (84.426)	-0.293	0.096
BMI (kg/m^2^)	159	96.9	5	3.1	46	28.0	31.9	5.71	113	68.9	25.62	4.79	*t* = −6.588 (72.067	-1.241	**0.001**
Time of blood assessment (h)	85	51.8	79	48.2	30	18.3	7.90	0.48	55	33.5	7.96	0.74	*t* = 0.477 (80.513)	0.096	0.634
Primary mediators of AL															
Cortisol (nmol/l)	157	95.7	7	4.3	44	26.8	340.90	132.98	113	68.9	410.13	144.63	*t* = 2.858 (84.825)	0.489	**0.005**
DHEA-S (*μ*mol/l)	158	96.3	8	3.7	46	28.0	3.58	2.14	112	68.3	4.02	2.08	*t* = 1.200 (81.804)	0.213	0.234
Epinephrine (pg/ml)	144	87.8	20	12.2	41	25.0	124.95	84.38	103	62.8	185.67	124.44	*t* = 3.373 (107.609)	0.530	**0.001**
Norepinephrine (pg/ml)	144	87.8	20	12.2	43	26.2	390.54	213.61	101	61.6	390.77	172.03	*t* = 0.009 (66.277)	0.002	0.993
Primary ALI	148	90.2	16	9.8	44	26.8	0.86	0.96	104	82.2	1.01	0.92	*t* = 1.264 (76.867)	0.233	0.210
Blood pressure															
Systolic (mmHg)	44	26.8	120	73.2	16	9.7	129.63	13.70	28	17.1	126.29	19.69	*t* = −0.660 (40.193)	-0.188	0.513
Diastolic (mmHg)	44	26.8	120	73.2	16	9.7	88.63	6.55	28	17.1	83.04	11.60	*t* = −2.043 (42.000)	-0.554	**0.047**
Lipid profile															
Triglycerides (mg/dl)	164	100	0	0.0	46	28.0	178.86	80.49	118	72.0	99.12	53.35	*t* = −6.209 (61.040)	-1.284	**0.001**
Cholesterol (mg/dl)	163	99.4	1	0.6	45	27.4	206.11	57.94	118	72.0	188.30	47.67	*t* = −1.838 (67.943)	-0.307	0.070
LDL-C (mg/dl)	164	100	0	0	46	28.0	125.33	44.17	118	72.0	115.28	36.15	*t* = −1.909 (69.746)	-0.362	0.060
HDL-C (mg/dl)	164	100	0	0	46	28.0	39.56	11.22	112	68.3	56.92	15.53	*t* = 7.942 (113.073)	1.201	**0.001**
Glycemia															
Fasting blood glucose (mg/dl)	157	95.7	7	4.3	45	27.4	103.82	33.40	113	68.9	82.74	8.00	*t* = −4.187 (46.042)	-1.107	**0.001**
Lipid medication	154	93.9	10	6.1	45	27.4	-	-	109	66.5	-	-	*χ* ^2 a^ = 4.908 (1)	-	0.084
Yes	2	1.2	-	-	2	1.2	-	-	0	0	-	-	-	-	
No	152	92.7	-	-	43	26.2	-	-	109	66.5	-	-	-	-	
Cholesterol medication	154	93.9	10	6.1	45	27.4	-	-	109	66.5	-	-	*χ* ^2 a^ = 0.051 (1)	-	1.000
Yes	6	3.6	-	-	2	1.2	-	-	4	2.4	-	-	-	-	
No	148	90.3	-	-	43	26.2	-	-	105	64.1	-	-	-	-	
Antidiabetic medication	154	93.9	10	6.1	45	27.4	-	-	109	66.5	-	-	*χ* ^2 a^ = 9.947 (1)	-	**0.007**
Yes	4	2.4	-	-	4	2.4	-	-	0	0	-	-	-	-	
No	150	91.5	-	-	41	25.0	-	-	109	66.5	-	-	-	-	
Hypertension medication	154	93.9	10	6.1	45	27.4	-	-	109	66.5	-	-	*χ* ^2^ = 24.123 (1)	-	**0.001**
Yes	43	26.2	-	-	25	15.2	-	-	18	10.9	-	-	-	-	
No	111	67.4	-	-	20	12.2	-	-	91	55.6	-	-	-	-	
Insulin medication	154	93.9	10	6.1	45	27.4	-	-	109	66.5	-	-	*χ* ^2 a^ = 4.908 (1)	-	0.084
Yes	2	1.2	-	-	2	1.2	-	-	0	0	-	-	-	-	
No	152	92.7	-	-	43	26.2	-	-	109	66.5	-	-	-	-	

Key: ^a^Fisher's exact test. BMI: body mass index; *M*: mean; SD: standard deviation; MD: mean difference; HDL-C: high-density lipoprotein cholesterol; LDL-C: low density lipoprotein cholesterol; DHEA-S: dehydroepiandrosterone sulfate; Primary ALI: primary allostatic load index. Significant differences *p* < 0.05 are shown in bold.

**Table 4:** Binary regression analysis on the association of primary mediators of allostatic load with metabolic syndrome in depressed patients.

Variable	Adjustment	*B*	Standard error	OR	95% CI		
					LL	UL	*p*
Cortisol	None	-0.004	0.001	0.996	0.994	0.996	**0.008**
	Age	-0.003	0.001	0.997	0.994	0.999	**0.018**
	Age, sex	-0.003	0.001	0.997	0.994	1.000	0.045
	Age, sex, BDI-II	-0.003	0.002	0.997	0.994	1.000	0.034

DHEA-S	None	-0.105	0.087	0.900	0.760	1.067	0.226
	Age	-0.015	0.097	0.985	0.815	1.191	0.878
	Age, sex	-0.082	0.104	0.921	0.752	1.129	0.429
	Age, sex, BDI-II	-0.120	0.108	0.887	0.718	1.095	0.265

Epinephrine	None	-0.005	0.002	0.995	0.991	0.999	**0.007**
	Age	-0.005	0.002	0.995	0.991	0.998	**0.006**
	Age, sex	-0.005	0.002	0.995	0.991	0.999	**0.008**
	Age, sex, BDI-II	-0.006	0.002	0.994	0.990	0.998	**0.007**

Norepinephrine	None	0.000	0.001	1.000	0.992	1.002	0.992
	Age	0.000	0.001	1.000	0.998	1.002	0.781
	Age, sex	0.000	0.001	1.000	0.998	1.002	0.755
	Age, sex, BDI-II	-0.001	0.001	0.999	0.997	1.001	0.382

Primary ALI	None	-0.270	0.210	0.198	0.505	1.152	0.198
	Age	-0.383	0.218	0.682	0.445	1.046	0.080
	Age, sex	-0.303	0.226	0.739	0.474	1.151	0.181
	Age, sex, BDI-II	-0.311	0.239	0.732	0.458	1.170	0.193

Key: DHEA-S: dehydroepiandrosterone sulfate; CI: confidence interval; LL: lower level; UL: upper level. Significant associations (*p* < 0.01) are shown in bold after adjusting for multiple comparisons using Bonferroni's correction.

## Data Availability

Data containing potentially identified or sensitive patient information is restricted by European law (GDPR). The data used in this study containing clinical participants is unavailable in a public repository. However, data are available upon reasonable request to Pia-Maria Wippert (wippert@uni-potsdam.de).

## References

[1] McEwen B. S. (2017). Allostasis and the epigenetics of brain and body health over the life course: the brain on stress. *JAMA Psychiatry*.

[2] Seeman T. E., Singer B. H., Rowe J. W., Horwitz R. I., McEwen B. S. (1997). Price of adaptation—allostatic load and its health consequences. *Archives of Internal Medicine*.

[3] Sterling P., Eyer J., Fisher S., Reason J. (1988). Handbook of Life Stress. *Handbook of Life Stress, Cognition and Health, January 1988*.

[4] McEwen B. S., Stellar E. (1993). Stress and the individual. *Archives of Internal Medicine*.

[5] de Kloet E. R., Sibug R. M., Helmerhorst F. M., Schmidt M. V. (2005). Stress, genes and the mechanism of programming the brain for later life. *Neuroscience and Biobehavioral Reviews*.

[6] Kirschbaum C., Pirke K. M., Hellhammer D. H. (2004). The 'Trier Social Stress Test'--a tool for investigating psychobiological stress responses in a laboratory setting. *Neuropsychobiology*.

[7] Seeley W. W., Menon V., Schatzberg A. F. (2007). Dissociable intrinsic connectivity networks for salience processing and executive control. *The Journal of Neuroscience: The Official Journal of the Society for Neuroscience*.

[8] Hermans E. J., van Marle H. J., Ossewaarde L. (2011). Stress-related noradrenergic activity prompts large-scale neural network reconfiguration. *Science*.

[9] Hermans E. J., Henckens M. J., Joëls M., Fernández G. (2014). Dynamic adaptation of large-scale brain networks in response to acute stressors. *Trends in Neurosciences*.

[10] Joëls M., Baram T. Z. (2009). The neuro-symphony of stress. *Neuroscience*.

[11] Sandner M., Lois G., Streit F. (2020). Investigating individual stress reactivity: high hair cortisol predicts lower acute stress responses. *Psychoneuroendocrinology*.

[12] Liu Y. Z., Wang Y. X., Jiang C. L. (2017). Inflammation: the common pathway of stress-related diseases. *Frontiers in Human Neuroscience*.

[13] Tian R., Hou G., Li D., Yuan T. F. (2014). A possible change process of inflammatory cytokines in the prolonged chronic stress and its ultimate implications for health. *The Scientific WorldJournal*.

[14] Beckie T. M. (2012). A systematic review of allostatic load, health, and health disparities. *Biological Research for Nursing*.

[15] Leahy R., Crews D. E. (2012). Physiological dysregulation and somatic decline among elders: modeling, applying and re-interpreting allostatic load. *Collegium Antropologicum*.

[16] O'Connor D. B., Thayer J. F., Vedhara K. (2021). Stress and health: a review of psychobiological processes. *Annual Review of Psychology*.

[17] Seeman T. E., McEwen B. S., Rowe J. W., Singer B. H. (2001). Allostatic load as a marker of cumulative biological risk: MacArthur studies of successful aging. *Proceedings of the National Academy of Sciences of the United States of America*.

[18] Kumar A., Rinwa P., Kaur G., Machawal L. (2013). Stress: neurobiology, consequences and management. *Journal of Pharmacy & Bioallied Sciences*.

[19] Plotsky P. M. (1987). Facilitation of immunoreactive corticotropin-releasing factor secretion into the hypophysial-portal circulation after activation of catecholaminergic pathways or central norepinephrine injection. *Endocrinology*.

[20] Carrasco G. A., Van de Kar L. D. (2003). Neuroendocrine pharmacology of stress. *European Journal of Pharmacology*.

[21] Van de Kar L. D., Blair M. L. (1999). Forebrain pathways mediating stress-induced hormone secretion. *Frontiers in Neuroendocrinology*.

[22] Hillhouse E. W., Grammatopoulos D. K. (2006). The molecular mechanisms underlying the regulation of the biological activity of corticotropin-releasing hormone receptors: implications for physiology and pathophysiology. *Endocrine Reviews*.

[23] Malta M. B., Martins J., Novaes L. S. (2021). Norepinephrine and glucocorticoids modulate chronic unpredictable stress-induced increase in the type 2 CRF and glucocorticoid receptors in brain structures related to the HPA axis activation. *Molecular Neurobiology*.

[24] De Kloet E. R., Derijk R. (2004). Signaling pathways in brain involved in predisposition and pathogenesis of stress-related disease: genetic and kinetic factors affecting the MR/GR balance. *Annals of the New York Academy of Sciences*.

[25] Su Y. A., Si T. (2022). Progress and challenges in research of the mechanisms of anhedonia in major depressive disorder. *General psychiatry*.

[26] Abdoli N., Salari N., Darvishi N. (2022). The global prevalence of major depressive disorder (MDD) among the elderly: a systematic review and meta-analysis. *Neuroscience and Biobehavioral Reviews*.

[27] Geer E. B., Islam J., Buettner C. (2014). Mechanisms of glucocorticoid-induced insulin resistance: focus on adipose tissue function and lipid metabolism. *Endocrinology and Metabolism Clinics of North America*.

[28] Osei F., Block A., Wippert P. M. (2022). Association of primary allostatic load mediators and metabolic syndrome (MetS): a systematic review. *Frontiers in Endocrinology*.

[29] Yu S., Guo X., Li G. X., Yang H., Zheng L., Sun Y. (2020). Metabolic syndrome associated with the onset of depressive symptoms among women but not men in rural Northeast China. *BMC Psychiatry*.

[30] Leonard B. E. (2010). The concept of depression as a dysfunction of the immune system. *Current Immunology Reviews*.

[31] Perrin A. J., Horowitz M. A., Roelofs J., Zunszain P. A., Pariante C. M. (2019). Glucocorticoid resistance: is it a requisite for increased cytokine production in depression? A systematic review and meta-analysis. *Frontiers in Psychiatry*.

[32] Yang L., Zhao Y., Wang Y. (2015). The effects of psychological stress on depression. *Current Neuropharmacology*.

[33] Herman J. P., McKlveen J. M., Ghosal S. (2016). Regulation of the hypothalamic-pituitary-adrenocortical stress response. *Comprehensive Physiology*.

[34] Anagnostis P., Athyros V. G., Tziomalos K., Karagiannis A., Mikhailidis D. P. (2009). The pathogenetic role of cortisol in the metabolic syndrome: a hypothesis. *The Journal of Clinical Endocrinology and Metabolism*.

[35] Faulconbridge L. F., Bechtel C. F. (2014). Depression and disordered eating in the obese person. *Current Obesity Reports*.

[36] Bremner J. D., Moazzami K., Wittbrodt M. T. (2020). Diet, stress and mental health. *Nutrients*.

[37] Marazziti D., Arone A., Palermo S. (2023). The wicked relationship between depression and metabolic syndrome. *Clinical Neuropsychiatry*.

[38] Alberti K., Eckel R., Grundy S. (2009). Harmonizing the metabolic syndrome: a joint interim statement of the International Diabetes Federation Task Force on Epidemiology and Prevention; National Heart, Lung, and Blood Institute; American Heart Association; World Heart Federation; International Atherosclerosis Society; and International Association for the Study of Obesity. *Circulation*.

[39] Block A., Schipf S., Van der Auwera S. (2016). Sex- and age-specific associations between major depressive disorder and metabolic syndrome in two general population samples in Germany. *Nordic Journal of Psychiatry*.

[40] Tyrka A. R., Walters O. C., Price L. H., Anderson G. M., Carpenter L. L. (2012). Altered response to neuroendocrine challenge linked to indices of the metabolic syndrome in healthy adults. *Hormone and metabolic research = Hormon- und Stoffwechselforschung = Hormones et metabolisme*.

[41] Vancampfort D., Correll C. U., Wampers M. (2014). Metabolic syndrome and metabolic abnormalities in patients with major depressive disorder: a meta-analysis of prevalences and moderating variables. *Psychological Medicine*.

[42] Almadi T., Cathers I., Chow C. M. (2013). Associations among work-related stress, cortisol, inflammation, and metabolic syndrome. *Psychophysiology*.

[43] Lee Z. S., Critchley J. A., Tomlinson B. (2001). Urinary epinephrine and norepinephrine interrelations with obesity, insulin, and the metabolic syndrome in Hong Kong Chinese. *Metabolism: Clinical and Experimental*.

[44] Lambert E. A., Lambert G. W. (2011). Stress and its role in sympathetic nervous system activation in hypertension and the metabolic syndrome. *Current Hypertension Reports*.

[45] Rasmusson A. M., Schnurr P. P., Zukowska Z., Scioli E., Forman D. E. (2010). Adaptation to extreme stress: post-traumatic stress disorder, neuropeptide Y and metabolic syndrome. *Experimental Biology and Medicine*.

[46] Gómez-Santos C., Hernández-Morante J. J., Tébar F. J., Granero E., Garaulet M. (2012). Differential effect of oral dehydroepiandrosterone‐sulphate on metabolic syndrome features in pre- and postmenopausal obese women. *Clinical Endocrinology*.

[47] Navar D., Saulis D., Corll C., Svec F., Porter J. R. (2006). Dehydroepiandrosterone (DHEA) blocks the increase in food intake caused by neuropeptide Y (NPY) in the Zucker rat. *Nutritional Neuroscience*.

[48] Hernandez-Morante J. J., Milagro F., Gabaldon J. A., Martinez J. A., Zamora S., Garaulet M. (2006). Effect of DHEA-sulfate on adiponectin gene expression in adipose tissue from different fat depots in morbidly obese humans. *European Journal of Endocrinology*.

[49] Boiko A. S., Mednova I. A., Kornetova E. G. (2020). Cortisol and DHEA-S related to metabolic syndrome in patients with schizophrenia. *Neuropsychiatric Disease and Treatment*.

[50] Carbone J. T. (2021). Allostatic load and mental health: a latent class analysis of physiological dysregulation. *Stress (Amsterdam, Netherlands)*.

[51] Hough C. M., Bersani F. S., Mellon S. H. (2021). Pre-treatment allostatic load and metabolic dysregulation predict SSRI response in major depressive disorder: a preliminary report. *Psychological Medicine*.

[52] Wippert P. M., Block A., Mansuy I. M. (2019). Alterations in bone homeostasis and microstructure related to depression and allostatic load. *Psychotherapy and Psychosomatics*.

[53] Beck A. T., Steer R. A., Brown G. (1996). Beck depression inventory–II. *Psychological assessment. Manual for the beck depression inventory-II*.

[54] Steer R. A., Rissmiller D. J., Beck A. T. (2000). Use of the Beck depression inventory-II with depressed geriatric inpatients. *Behaviour Research and Therapy*.

[55] Herzberg P. Y., Goldschmidt S., Heinrichs N. (2008). Beck depressions-inventar (BDI-II). Revision. *Report Psychologie*.

[56] Kühner C., Bürger C., Keller F., Hautzinger M. (2007). Reliabilität und Validität des revidierten Beck-Depressionsinventars (BDI-II). *Der Nervenarzt*.

[57] Strazzullo P., Barbato A., Siani A. (2008). Diagnostic criteria for metabolic syndrome: a comparative analysis in an unselected sample of adult male population. *Metabolism: Clinical and Experimental*.

[58] McEwen B. S., Rasgon N. L., Strain J. J., Blumenfield M. (2018). The brain and body on stress: allostatic load and mechanisms for depression and dementia. *Depression as a Systemic Illness*.

[59] Capuron L., Su S., Miller A. H. (2008). Depressive symptoms and metabolic syndrome: is inflammation the underlying link?. *Biological Psychiatry*.

[60] Laudisio A., Marzetti E., Pagano F., Pozzi G., Bernabei R., Zuccalà G. (2009). Depressive symptoms and metabolic syndrome: selective association in older women. *Journal of Geriatric Psychiatry and Neurology*.

[61] Constantinopoulos P., Michalaki M., Kottorou A. (2015). Cortisol in tissue and systemic level as a contributing factor to the development of metabolic syndrome in severely obese patients. *European Journal of Endocrinology*.

[62] Park S. B., Blumenthal J. A., Lee S. Y., Georgiades A. (2011). Association of cortisol and the metabolic syndrome in Korean men and women. *Journal of Korean Medical Science*.

[63] Garcez A., Leite H. M., Weiderpass E. (2018). Basal cortisol levels and metabolic syndrome: a systematic review and meta-analysis of observational studies. *Psychoneuroendocrinology*.

[64] McEwen B. S., Gianaros P. J. (2011). Stress- and allostasis-induced brain plasticity. *Annual Review of Medicine*.

[65] Myers B., McKlveen J. M., Herman J. P. (2012). Neural regulation of the stress response: the many faces of feedback. *Cellular and Molecular Neurobiology*.

[66] Kleshchova O., Weierich M. R. (2021). The neurobiology of stress. *Biopsychosocial Factors of Stress, and Mindfulness for Stress Reduction*.

[67] de Punder K., Pruimboom L. (2015). Stress induces endotoxemia and low-grade inflammation by increasing barrier permeability. *Frontiers in Immunology*.

[68] Monteiro R., Azevedo I. (2010). Chronic inflammation in obesity and the metabolic syndrome. *Mediators of Inflammation*.

[69] Pahwa R., Goyal A., Jialal I. (2023). Chronic inflammation. *StatPearls*.

[70] Heindel J. J., Blumberg B., Cave M. (2017). Metabolism disrupting chemicals and metabolic disorders. *Reproductive Toxicology*.

[71] Xu C., He J., Jiang H. (2009). Direct effect of glucocorticoids on lipolysis in adipocytes. *Molecular Endocrinology*.

[72] Heim C., Ehlert U., Hellhammer D. H. (2000). The potential role of hypocortisolism in the pathophysiology of stress-related bodily disorders. *Psychoneuroendocrinology*.

[73] Maripuu M., Wikgren M., Karling P., Adolfsson R., Norrback K. F. (2016). Relative hypocortisolism is associated with obesity and the metabolic syndrome in recurrent affective disorders. *Journal of Affective Disorders*.

[74] Ebert S. N., Rong Q., Boe S., Thompson R. P., Grinberg A., Pfeifer K. (2004). Targeted insertion of the Cre-recombinase gene at the phenylethanolamine n-methyltransferase locus: a new model for studying the developmental distribution of adrenergic cells. *Developmental dynamics: an official publication of the American Association of Anatomists*.

[75] Lindmark S., Lönn L., Wiklund U., Tufvesson M., Olsson T., Eriksson J. W. (2005). Dysregulation of the autonomic nervous system can be a link between visceral adiposity and insulin resistance. *Obesity Research*.

[76] Delahanty D. L., Nugent N. R., Christopher N. C., Walsh M. (2005). Initial urinary epinephrine and cortisol levels predict acute PTSD symptoms in child trauma victims. *Psychoneuroendocrinology*.

[77] Velmurugan B. K., Baskaran R., Huang C. Y. (2019). Detailed insight on *β*-adrenoceptors as therapeutic targets. *Biomedicine & Pharmacotherapy = Biomedecine & Pharmacotherapie*.

[78] Klinge C. M., Clark B. J., Prough R. A. (2018). Dehydroepiandrosterone research: past, current, and future. *Vitamins and Hormones*.

[79] Goldman N., Turra C. M., Glei D. A., Lin Y. H., Weinstein M. (2006). Physiological dysregulation and changes in health in an older population. *Experimental Gerontology*.

[80] Sun J., Wang S., Zhang J.-Q., Li W. (2007). Assessing the cumulative effects of stress: the association between job stress and allostatic load in a large sample of Chinese employees. *Work & Stress*.

[81] Black P. H. (1994). Immune system-central nervous system interactions: effect and immunomodulatory consequences of immune system mediators on the brain. *Antimicrobial Agents and Chemotherapy*.

[82] Osimo E. F., Pillinger T., Rodriguez I. M., Khandaker G. M., Pariante C. M., Howes O. D. (2020). Inflammatory markers in depression: a meta-analysis of mean differences and variability in 5, 166 patients and 5, 083 controls. *Brain, Behavior, and Immunity*.

[83] Qiu W., Cai X., Zheng C., Qiu S., Ke H., Huang Y. (2021). Update on the relationship between depression and neuroendocrine metabolism. *Frontiers in Neuroscience*.

[84] Juster R. P., McEwen B. S., Lupien S. J. (2010). Allostatic load biomarkers of chronic stress and impact on health and cognition. *Neuroscience and Biobehavioral Reviews*.

[85] Stuchtey F. C., Block A., Osei F., Wippert P. M. (2022). Lipid biomarkers in depression: does antidepressant therapy have an impact?. *Healthcare*.

[86] Bot M., Milaneschi Y., Al-Shehri T. (2020). Metabolomics profile in depression: a pooled analysis of 230 metabolic markers in 5283 cases with depression and 10, 145 controls. *Biological Psychiatry*.

[87] Fava M. (2000). Weight gain and antidepressants. *The Journal of Clinical Psychiatry*.

[88] Shimizu S., Akiyama T., Kawada T. (2010). In vivo direct monitoring of interstitial norepinephrine levels at the sinoatrial node. *Autonomic Neuroscience: Basic & Clinical*.

[89] Behr G. A., Moreira J. C., Frey B. N. (2012). Preclinical and clinical evidence of antioxidant effects of antidepressant agents: implications for the pathophysiology of major depressive disorder. *Oxidative Medicine and Cellular Longevity*.

[90] Cohen H. W., Gibson G., Alderman M. H. (2000). Excess risk of myocardial infarction in patients treated with antidepressant medications: association with use of tricyclic agents. *The American Journal of Medicine*.

[91] McIntyre R. S., Soczynska J. K., Konarski J. Z., Kennedy S. H. (2006). The effect of antidepressants on lipid homeostasis: a cardiac safety concern?. *Expert Opinion on Drug Safety*.

[92] Moazzami K., Lima B. B., Sullivan S., Shah A., Bremner J. D., Vaccarino V. (2019). Independent and joint association of obesity and metabolic syndrome with depression and inflammation. *Health psychology: official journal of the Division of Health Psychology, American Psychological Association*.

[93] Tomiyama A. J., Hunger J. M., Nguyen-Cuu J., Wells C. (2016). Misclassification of cardiometabolic health when using body mass index categories in NHANES 2005-2012. *International Journal of Obesity (2005)*.

[94] Elder G. J., Ellis J. G., Barclay N. L., Wetherell M. A. (2016). Assessing the daily stability of the cortisol awakening response in a controlled environment. *BMC Psychology*.

[95] Bowles N. P., Thosar S. S., Butler M. P. (2022). The circadian system modulates the cortisol awakening response in humans. *Frontiers in Neuroscience*.

[96] Cheng J., Ju S., Zhang Z. (2023). Osteoporotic vertebral compression fractures caused by Cushing's syndrome in young women: case report and literature review. *BMC Musculoskeletal Disorders*.

[97] Dedovic K., Ngiam J. (2015). The cortisol awakening response and major depression: examining the evidence. *Neuropsychiatric Disease and Treatment*.

[98] Linnemann P., Friedrich N., Nauck M., Teismann H., Berger K. (2023). The relationship between cortisol awakening response and trait resilience in two patient cohorts and one population-based cohort. *The World Journal of Biological Psychiatry*.

[99] Wardenaar K. J., Vreeburg S. A., van Veen T. (2011). Dimensions of depression and anxiety and the hypothalamo-pituitary-adrenal axis. *Biological Psychiatry*.

[100] El-Farhan N., Rees D. A., Evans C. (2017). Measuring cortisol in serum, urine and saliva - are our assays good enough?. *Annals of Clinical Biochemistry*.

[101] Richard V. R., Zahedi R. P., Eintracht S., Borchers C. H. (2020). An LC-MRM assay for the quantification of metanephrines from dried blood spots for the diagnosis of pheochromocytomas and paragangliomas. *Analytica Chimica Acta*.

